# Metabolic Perturbation and Synthetic Biology Strategies for Plant Terpenoid Production—An Updated Overview

**DOI:** 10.3390/plants10102179

**Published:** 2021-10-14

**Authors:** Vimalraj Mani, Soyoung Park, Jin A Kim, Soo In Lee, Kijong Lee

**Affiliations:** Department of Agricultural Biotechnology, National Institute of Agricultural Sciences, Rural Development Administration, Jeonju 54874, Korea; vimalraj08@gmail.com (V.M.); psy0203@korea.kr (S.P.); jakim72@korea.kr (J.A.K.); silee@korea.kr (S.I.L.)

**Keywords:** metabolic engineering, synthetic biology, linalool, costunolide, FPP-farnesyl pyrophosphate, GPP-geranyl pyrophosphate

## Abstract

Terpenoids represent one of the high-value groups of specialized metabolites with vast structural diversity. They exhibit versatile human benefits and have been successfully exploited in several sectors of day-to-day life applications, including cosmetics, foods, and pharmaceuticals. Historically, the potential use of terpenoids is challenging, and highly hampered by their bioavailability in their natural sources. Significant progress has been made in recent years to overcome such challenges by advancing the heterologous production platforms of hosts and metabolic engineering technologies. Herein, we summarize the latest developments associated with analytical platforms, metabolic engineering, and synthetic biology, with a focus on two terpenoid classes: monoterpenoids and sesquiterpenoids. Accumulated data showed that subcellular localization of both the precursor pool and the introduced enzymes were the crucial factors for increasing the production of targeted terpenoids in plants. We believe this timely review provides a glimpse of current state-of-the-art techniques/methodologies related to terpenoid engineering that would facilitate further improvements in terpenoids research.

## 1. Introduction

Terpenoids are the most chemically, physically, and functionally complex family of natural chemicals found in living creatures, with over 80,000 compounds identified to date, and many more expected to exist [1]. They are the most structurally varied group of plant-derived natural compounds, and are economically significant due to their use in a variety of industrial products, including pharmaceuticals, flavoring agents, insecticides, antimicrobial agents, and perfumes [2]. They play an important role in plant–environment, plant–plant, plant–insect, and plant–animal interactions in nature [3]. Many terpenoids have a strong link to primary metabolism (e.g., phytol, the plant hormone gibberellin, and carotenoid pigments), while others are common secondary metabolites in plants [4].

Terpene biochemistry and chemistry have been studied for over a century, mainly in plants [5]. Recently, genes that encode enzymes and regulators engaged in terpene biosynthesis have been discovered, and their genomic location, mode of expression, and long-term evolution have been investigated [6]. Despite their considerable structural variations, all terpenoids are derived from the universal isoprene C5 building blocks. The terpenoid backbone is synthesized from two precursors: IPP (isopentenyl pyrophosphate), and its isomer dimethylallyl pyrophosphate (DMAPP). Terpene biosynthesis is a complex mechanism involving two independent biosynthetic pathways. The cytosolic mevalonate (MVA) pathway is found in most eukaryotes (all mammals, the cytosol and mitochondria of plants, fungi), archea, and some bacteria [7,8]. They are utilized in the production of bigger compounds, such as sesquiterpenes (C15), triterpenes (C30), sterols (C27–29), and dolichols (C40–50). The methylerythritol phosphate (MEP) pathway is found in plant chloroplasts, bacteria, algae, and cyanobacteria. This pathway predominantly produces mono-terpenes (C10), diterpenes (C20), and tetraterpenes (C40). Unlike most microbial organisms, both pathways operate in plants—the MEP pathway in the chloroplast and the MVA pathway in the cytoplasm—and the labor division between them represents a complex array of chemicals that control the development and growth of plants, and interact with plants and their surroundings to control these interactions [9].

Over recent years, efforts to generate large amounts of monoterpenes and sesquiterpenes in transgenic plants proved effective. Many plant species have been genetically modified, with the overexpression of terpene synthase under constitutive promoters being the most common. Plants with overexpressed linalool synthase genes that were produced include tomato, petunia, *Arabidopsis*, potato, and carnation [10,11,12,13,14]. Such plants generated and released linalool and its glycosylated or hydroxylated derivatives. Similarly, *α*-pinene, *γ*-terpinene, and limonene synthases were shown to alter the terpenoid profile of tobacco and mint plants [15]. The overexpression of gene encoding enzymes from various stages of the MEP pathway may result in even higher levels of terpenoid precursors (DXR and HDR) [16]. The genes encoding enzymes that modify the monoterpene composition have also been effectively overexpressed or knocked down in mint and tobacco. Sesquiterpene production in transgenic plants is more difficult than monoterpene production. In an effort to engineer sesquiterpenes in plants using terpene synthases, tobacco plants were transformed with *Artemisia annua* amorpha-4,11-diene synthase and fungal trichodiene synthase on either side, but this resulted in only a low-level yield [17,18].

Owing to their traditionally known pharmacological importance, terpenoids have at-attracted attention from plant breeders, biochemists, and pharmacologists, who have exploited them for their diverse metabolite/chemical profile. Several attempts have been made to decode their distinct metabolic profiles through high throughput metabolic fingerprinting methods including nuclear magnetic resonance (NMR), gas chromatography-mass spectrometry (GC-MS), liquid chromatography-mass spectrometry (LC-MS), and capillary electrophoresis-mass spectrometry (CE-MS), and so-called metabolomics [19]. Metabolic engineering is an appropriate system to either enhance or manipulate the synthesis of terpenes in plant species that naturally produce them, or to integrate terpene biosynthetic pathways into certain plants [20]. A number of attempts have been made over the last decade to engineer the production of monoterpenoid and sesquiterpenoid compounds in various plant species and tissues. Implementing modern analytical techniques is the best way to improve the qualitative and quantitative aspects of terpenes and terpenoids in plants and other products [21]. 

All plants produce a variety of terpenoid compounds that serve as phytohormones and anti-oxidants, or have other functions. Hundreds of distinct terpenoids are synthesized by different plant lineages, with the overall number of such advanced plant terpenoids estimated to be in the thousands [22]. A clear understanding of a plant’s chemical composition enables a more accurate assessment of its medicinal potential. Modern chemistry can unravel the primary metabolic functions of plants, including cell division, development, respiration, storage, and reproduction. It helps improve our understanding of process components, such as glycolysis, the cancer or cycles of citric acid, and photosynthesis [23]. Small molecules, such as sugars, amino acids, proteins, nucleic acids, and polysaccharides, are examples of primary metabolites [24,25]. Secondary plant metabolites are a diverse group of chemical compounds synthesized by the plant cell through metabolic pathways derived from primary metabolic pathways [26]. By the mid-twentieth century, advancements in analytical techniques such as GC-MS, LC-MS, CE-MS, and NMR enabled the recovery of an increasing number of these molecules, providing a basis for the development of phytochemistry. Secondary plant metabolites are classified into specific categories: phenolics, alkaloids, saponins, terpenes, lipids, and carbohydrates [27]. Terpenes are the major and most varied category of secondary metabolites found in plants. Green plants, especially flowering plants, have an extraordinarily high number of terpenoids compared to other living organisms [22]. Isoprene units in the molecules, mono-terpenoids (C10), sesquiterpenoids (C15), diterpenoids (C20), and triterpenoids (C30) are considered secondary metabolites. Many terpenoids are commercially interesting because of their use as flavors and fragrances in foods and cosmetics (e.g., menthol, nootkatone, and sclareol) or because they are important for the quality of agricultural products, for example, with the flavor of fruits and the fragrance of flowers (e.g., linalool) [28]. In addition, terpenoids have medicinal properties including anti-carcinogenic (e.g., taxol and perilla alcohol), antimalarial (e.g., artemisinin), anti-ulcer, hepaticidal, antimicrobial, and diuretic (e.g., glycyrrhizin) activities [29]. Terpenoids have also been shown to be of ecological significance. Compounds, such as the bitter triterpenoid cucurbitacins and the pungent diterpenoid polygodial, have been shown to be involved in insect resistance [30]. Other terpenoid substances are active in plant interactions, plant–microorganism interactions, and plant–arthropod herbivore interactions (for example, spider mite feeding induces (E, E)-a-farnesene in cucumber) [31]. In this review, we provide an overview of the recent developments in relation to different biosynthetic and regulatory aspects of monoterpenoid and sesquiterpenoid metabolism in plants. Moreover, we report on the remarkable improvement in metabolic engineering for advanced terpenoid production in various platforms.

## 2. Biosynthesis and Precursors of Terpenoids

**Figure 1:**
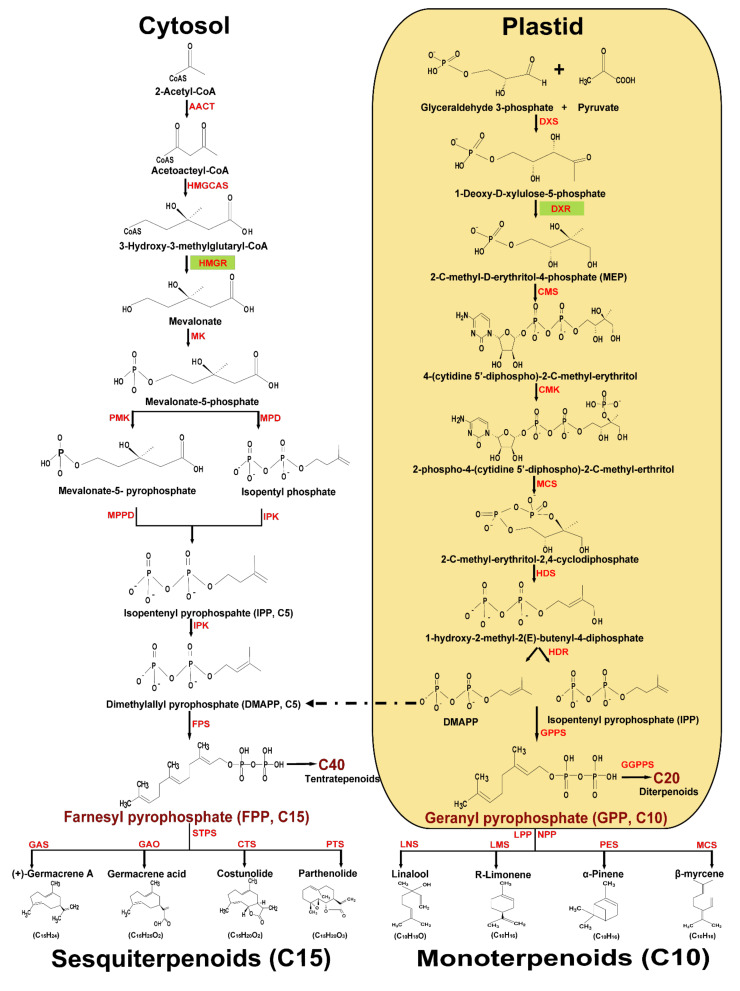
Terpenoid biosynthesis in plants. There are two distinct pathways in plants for the synthesis of the universal precursors isopentenyl pyrophosphate (IPP) and dimethyl-allyl pyrophosphate (DMAPP): the cytoplasm-localized mevalonate (MVA) pathway and the plastid-localized methyl erythritol phosphate (MEP) pathway. The brown color indicates intermediate precursors of plastids (geranyl pyrophosphate) and cytosol (farnesyl pyrophosphate). AACT acetoacetyl-CoA thiolase; HMGCAS 3-hydroxy-3-methylglutaryl-CoA synthase; HMGR 3-hydroxy-3-methylglutaryl-CoA reductase; MK mevalonate-5-kinase; PMK phosphomevalonate kinase; MPD mevalonate-5-phpshate decarboxylase; MPPD mevalonate pyrophosphate decarboxylase; IPK isopentyl pyrophosphate kinase; FPS farnesyl pyrophosphate synthase. DXS 1-deoxy-d-xylulose-5-phosphate synthase; DXR 1-deoxy-d-xylulose 5-phosphate reductoisomerase; CMS 2-c-methyl-d-erythritol 4-phosphate cytidylyltransferase; CMK 4-diphosphocytidyl-2-c-methyl-d-erythritol kinase; MCS 2-c-methyl-d-erythritol 2, 4-cyclodiphosphate synthase; HDS 4-hydroxy-3-methyl-but-2-enyldiphosphate synthase; HDR 4-hydroxy-3-methyl-but-2-enyldiphosphate reductase; GPPS geranyl pyrophosphate synthase; GGPPS geranylgeranyl pyrophosphate synthase; STPS sesquiterpene synthase; LPP linalyl pyrophosphate; NPP neryl pyrophosphate; GAO germacrene A oxidase; GAS germacrene A synthase; CTS costunolide synthase; PTS parthenolide synthase; LNS linalool synthase; LMS limonene synthase; PES pinene synthase; MCS myrcene synthase. As representative examples of terpenoids: linalool, limonene, α-pinene, β-myrcene, germacrene A, germacrene A acid, costunolide and parthenolide are illustrated in chemical structure.

Plants possess two distinct pathways to produce terpenoids: the plastidial 2-c-methyl-d-erythritol-4-phosphate (MEP) pathway and the acetyl-CoA dependent cytosolic mevalonate (MVA) pathway (Figure 1). The C5 unit’s IPP (isopentenyl pyrophosphate) and its allylic isomer dimethylallyl pyrophosphate (DMAPP), the fundamental terpenoid biosynthesis building blocks, are produced through a metabolic assembly of multiples in both terpenoids pathways. Prenyl transferases use DMAPP and IPP in condensation reactions to generate bigger prenyl diphosphates, including the sesquiterpene precursor FPP (farnesyl pyrophosphate), monoterpene precursor GPP (geranyl pyrophosphate), and C40 carotenoid and diterpene precursor GGPP (geranylgeranyl pyrophosphate) in both compartments (Figure 1). Although there is increasing evidence that there is an exchange of intermediates between these compartments [32,33], the cytoplasmic MVA pathway is generally considered to supply the precursors for the production of sesquiterpenes. In the plastids, the MEP pathway supplies the precursors for the production of monoterpenes (Figure 1). The MVA pathway consists of seven enzymatic processes that convert the precursor acetyl-CoA to IPP and DMAPP (Figure 1). The MEP pathway requires eight enzymatic steps to convert the initial materials pyruvate and glyceraldehyde-3-phosphate to IPP and DMAPP (Figure 1). Even though IPP and DMAPP are separated spatially, they are exchanged between cytosol and plastids during the production of various terpenoids [34]. Using precursors such as GPP, FPP and GGPP are cyclized or rearranged by different terpene synthase enzymes, and are responsible for the synthesis of different classes of terpenoids; they can easily acquire new catalytic properties by minor changes in the structure [35]. 

Monoterpenoids are C10 compounds derived from GPP through the enzymatic activity of geranyl pyrophosphate synthase (GPS). Through the cyclization process, the range of monoterpenes is rapidly increased, and monocyclic or bicyclic compounds can be synthesized. The dephosphorylation and ionization of geranyl diphosphate to geranyl carbo-cation initiates the production of monoterpenes [36]. Linalyl pyrophosphate and neryl pyrophosphate are isomers of GPP by ionization to the allylic cation. This allows for changes in the attachment of the diphosphate group or changes in stereochemistry at the double bond. Monoterpene synthase (or monoterpene cyclase) is an enzyme that catalyzes the formation of cyclic monoterpenoids. There is an essential enzyme involved in the synthesis of each monoterpenoids, such as linalool synthase for linalool, limonene synthase for limonene, pinene synthase for pinene, and myrcene synthase for myrcene [37,38] (Figure 1).

C15 sesquiterpenoids are derived from FPP by the action of farnesyl pyrophosphate synthase (FPS), and is the common precursor of all sesquiterpenoid lactones (STLs). Sesquiterpene synthase (STPS) catalyzes the cyclisation of FPP in the first step of STL biosynthesis [39]. They are mainly located in the cytosol and are characterized by their plasticity, showing the capacity of multiple substrate utilization. Germacrene A synthase (GAS) is one of the best-characterized STPS, converting FPP into germacrene A (GA). Germacrene A oxidase (GAO), a cytochrome P450-like enzyme, converts GA into germacrene A acid (GAA). To produce costunolide, GAA is further oxidized by the costunolide synthase. Furthermore, to produce the end product of parthenolide, this costunolide catalyzes the epoxidation of the C4-C5 double bond. Figure 1 depicts the overall biosynthetic pathway and intermediate chemical structure of monoterpenoids and sesquiterpenoids [40].

### 2.1. Monoterpenoid Chemical Compounds

Monoterpenoids are substances that can be found in essential oils derived from different plants, including vegetables, fruits, herbs, and spices [41,42,43]. Monoterpenes are C10 molecules that can be acyclic, monocyclic, or bicyclic. Monoterpene synthase uses GPP (geranyl pyrophosphate) as a substrate to produce them. Additionally, GPP is also a substrate for the production of GGPP (geranyl-geranyl pyrophosphate) and FPP (farnesyl pyrophosphate), two important substances in animal, plant, and yeast cell metabolism [22]. Monoterpenoid compounds are classified as acyclic (e.g., linalool, geraniol, β-myrcene, (+)-citronellol, nerol), monocyclic (thymol, (−)-menthol, limonene, eugenol, γ-terpinene, terpinolene, and piperitone), bicyclic (α-pinene, (−)-β-pinene, camphene, sabinene, myrtenol, (+)-camphor, (−)-borneol, (+)-cis-verbenol, ∆3-terpinene, eucalyptol, sabinene hydrate, and fenchone), and others (α-phellandrene, ρ-cymene, ocimene, fenchol, (−)-isopulegol terpinen-4-ol, α-terpineol, (+)-dihydrocarvone, pulegone, carvone, geranyl acetate, methol-isomer, and safranal) [44]. Monoterpenoids are found mostly in the taxonomic groups Asteraceae, Apiaceae, Verbenaceae, Poaceae, Myrtaceae, Lamiaceae, Pinaceae, Rutaceae, Lauraceae, and Cannabaceae. The following monoterpenoid compounds are significant in plant species: α- and β- pinene (*Pinus palustris*), δ-3-carene, α-phellandrene, and myrcene (*Lippia citriodora*) are found as complex mixtures in most essential oils, particularly in those extracted from plant leaves, while seed and flower oils contain more specialized monoterpenes and present fruity or flowery odors. Linalool has two stereoisomers are present: (*R*)-(−)-linalool and (*S*)-(+)-linalool. *S*-linalool is found in major constituents of the essential oils of coriander (*Coriandrum sativum* L.) seed, palmarosa (*Cymbopogon martinii* var martinii (Roxb.) Wats), and sweet orange (*Citrus sinensis* Osbeck) flowers. Meanwhile, (*R*)-Linalool is present in Ho oils from *Cinnamomun camphora*, rosewood oil, lavender (*Lavandula officinalis* Chaix), laurel (*Laurus nobilis*), and sweet basil (*Ocimum basilicum*) [2]. D-carvone from caraway (*Carum carvi*), with its spicy and bread-like fragrance; menthol is derived from wild mint (*Mentha arvensis*) and has a strong minty aroma; D-limonene from citrus species with a fresh orange peel odor; citral from lemongrass (*Cymbopogon citratus*) having a fresh lemon peel odor; Eucalyptol, also known as 1,8-cineole from eucalyptus (*Eucalyptus globulus*) having a camphoraceous cool odor. Menthone and isomenthone are components of essential oils such as pennyroyal, peppermint, *Pelargonium geraniums*, and others. Thymol (*Thymus vulgaris*); camphor derived from camphor tee (*Cinnamomun camphora*) [45].

### 2.2. Sesquiterpenoid Chemical Compounds 

Sesquiterpenoids are C15 compounds with three isoprene (C_5_H_8_) units that are primarily present in fresh raw plant materials. They form the most diverse terpenoids group, and the biosynthesis of sesquiterpenoids is synthesized using mevalonic acid [46,47,48]. There are about 150 known sesquiterpenes compounds including artemisinin, (−)-β-elemene, β-caryophyllene, aromaadendrene, trans-β-farnesene, α-humulene, valencene, ledene, trans-nerollidol, caryophyllene oxide, globulol, viridiflorol, (−)-guaiol, (+)-cedrol, β-eudesmol, α-bisabolol, *cis*-muurola-4(15), 5-diene, germacrene D, costunolide, parthenolide, guatterin A, dihydromadolin, madolin-K, madolin-W, malayscaphiol, sarcanolides A and B, perovskanol, eudesmane-type I and II, chrysanolide A, isocyperotun-done, and 1,4-epoxy-4-hydroxy-4-5-seco-guain-11-en-5-1 [49]. Sesquiterpenoids are predominantly found in the taxonomic groups Poaceae, Solanaceae, Araceae, Rutaceae, Zingiberaceae, Cannabaceae, Myrtaceae, and Cupressaceae. However, they are most common in the Asteraceae family, where they are almost ubiquitous. Sesquiterpenoid compounds are associated with the following plant species: Farnesol (*Cymbopogon* species), β-nerolidol (*Citrus aurantium*), α-humlene (*Humulus lupulus*), Zingiberene (*Zingiber officinale*), β-Santol (*Santalum album*), artemisinin (*Artemisia annua*), nootkaton (*Citrus paradisi*), costunolide (*Saussurea costus*), and parthenolide (*Rosmarinus officinalis*) [50].

## 3. Analytical Platforms for Terpenoids

### 3.1. Chromatographic Techniques

A number of methods used for analyzing terpenoids have already been developed; the most common approach is chromatographic analysis, specifically GC-MS or LC-MS. Compounds of monoterpenoids and sesquiterpenoids were determined by various methods such as GC-MS, GC-MS/MS, GC-FID (GC-Flame Ionization Detection), and GC-GC [51]. Each method has its own advantages and disadvantages. The main advantage of using GC-MS is that it is sensitive and robust, and also capable of routinely and reproducibly measuring hundreds of analytes across thousands of samples. Among the different methods, the best one for analyzing terpenes is solvent extraction followed by GC-FID analysis. The FID is a powerful instrument for quality control because of its low cost, accuracy, and simple interface. But the main disadvantage is that it provides little information other than the retention time. For better identification and characterization, various types of columns, dimensions, and oven programs were used (Table 1) [52,53,54,55,56,57,58,59]. Despite this, the optimum GC detector for terpene analysis is still unknown. The column ZB-5 PLUS^TM^ is used selectivity for high temperature limitations, allowing for a high resolution of essential terpenoids. Phenomenex TM groups that used this column in GC-FID identified 33 cannabis-derived primary and secondary terpenes [56]. Another reliable method for determining terpenoids is HS-GC-FID. Headspace sampling is a technique that involves heating a solid or liquid sample inside a sealed vial (which converts the volatile substance to the gas phase). This approach increases column lifetime and minimizes inlet maintenance by preventing nonvolatile material from entering the GC system. Apart from the aforementioned approaches, the use of GC-MS, another frequently used technique, has the additional advantage of spectral peak identification to ensure that selection is accurate. However, it may not be the optimal detector for terpene analysis due to the structural and functional similarity of terpene class molecules. Moreover, GC-MS provides a different level of sensitivity. GC-MS with high-temperature headspace sampling was used to quantify selected terpenoids using a TG-624 SilMS column [53]. The separated constituents were tentatively identified by comparing their mass spectra with those in the available MS library such as: Wiley, Flavors and Fragrances of Natural and Synthetic Compounds (FFNSC) and NIST08 (National Institute of Standards and Technology, Gaithersburg, MD, USA) and by comparing their retention indices (RIs) with literature values. Each constituent was quantified based on the comparison of its peak area with that of the internal standard, and the contents are expressed as ng/g FW. Despite the advantages of various GC methods, the main disadvantage is that degraded chemicals cannot be quantified effectively. In this case, researchers prefer LC-MS/MS for the characterization of degraded chemicals such as GPP and FPP. It is challenging to isolate these compounds using HPLC due to the ionic nature of the phosphate groups. MS, on the other hand, has an adequate sensitivity and specificity. These metabolites have been determined using HPLC-tandem MS (MS/MS) and ultra HPLC-MS/MS [60,61]. Furthermore, obtaining the proper analytical result of terpene analysis is a difficult task that necessitates the evaluation of a number of critical factors, such as equipment selection, instrument parameters, and the optimization of extraction methods.

**Figure 2:**
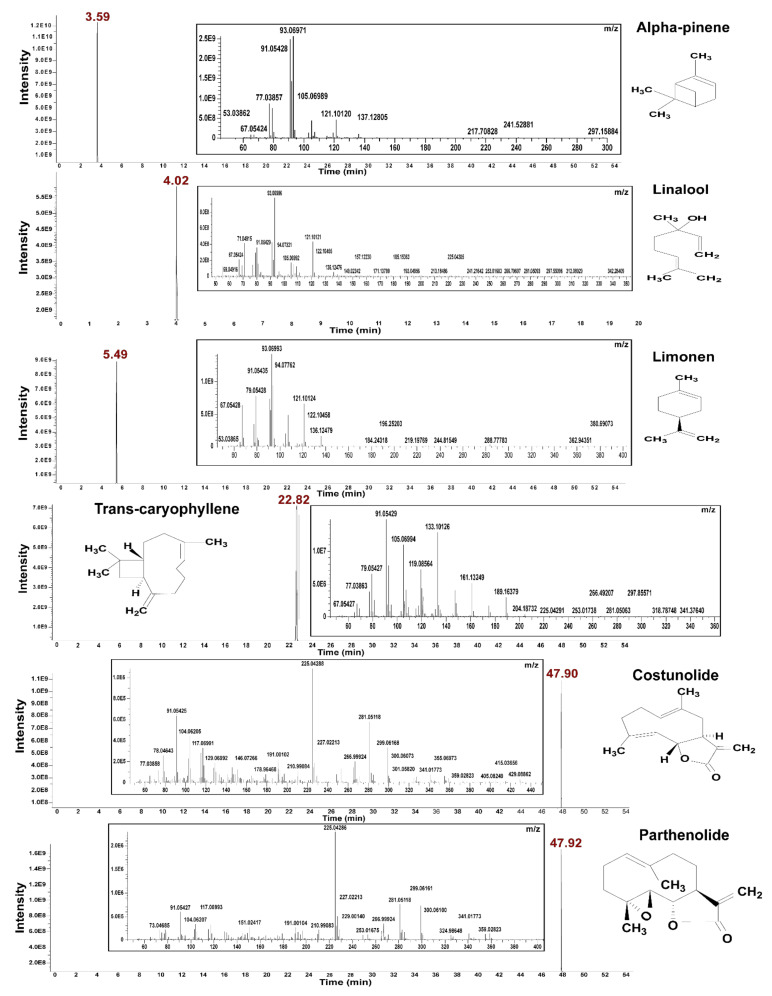
GC-Q-Orbitrap-MS profiling of three major standard compounds from monoterpenoids (alpha-pinene, linalool and limonene) and sesquiterpenoids (trans-caryophyllene, costunolide and parthenolide).

The general process of GC-MS analysis includes the injections of extracted compounds directly into the GC, and the separation of different components using capillary columns or similar columns of 30m in length or greater. Helium (99.99%), the carrier gas, will continuously pass through at a flow rate of 1 mL/min. Throughout all GC strokes, the oven temperature is typically set to begin at 60 °C and then ramped up to 160 °C (at an increasing rate of 5−10 °C/min). Following this initial stage, the temperature is often raised to 360 °C in order to elute the chemicals. Electron ionization (EI) sources were used in the GC-MS setup, with collision energies ranging from 15 to 70 eV depending on the target molecules. Total GC analysis time ranged from 2 to 50 min. Despite the similarity of several terpenes MS spectra, further identification was performed using the retention index. The retention indices for the chemicals detected in each sample were determined using an n-alkane standard analyzed on the same GC–MS instrument under identical GC conditions [52,53,54,55,56,57,58,59]. Here, metabolic profiles of monoterpenoid (linalool, limonene, and alpha-pinene) and sesquiterpenoid (costunolide, parthenolide, and trans-caryophyllene) groups, injected individually at a concentration of 100 µg/mL in GC-Q-Orbitrap-MS, are shown in Figure 2.

### 3.2. Metabolic Profiling of Plant Terpenoids 

In classic plant biotechnology, the metabolomic approach is generally used for metabolic profiling of a target set of metabolites, such as fatty acids, alkaloids, terpenes, phenolics, etc., in plant physiology and genetic research, along with other tools including genomics, transcriptomics, and proteomics [62]. Different analytical approaches were employed to determine the metabolomic compounds, and were utilized to estimate targeted metabolites such as NMR, GC-MS, LC-MS, and CE-MS. In plant extracts, there are no specific chemical technologies for the identification of monoterpenoids and sesquiterpenoids, unlike for other secondary metabolites such as triterpenes, carotenoids, phytosterols, flavonoids, and other primary metabolites [63]. Volatile compounds can be classified as endogenous or emitted. Solvents such as hexane, pentane, diethyl ether, dichloromethane, chloroform, ethyl acetate, and solvent mixtures can be used to extract endogenous volatiles. The use of an organic solvent has the advantage of reducing the activation of enzymes, which may alter the original composition of volatile compounds. A solid-phase extraction (SPE) column has been used in some cases to remove nonvolatile compounds from the organic solvent extracts. On the other hand, static headspace sampling techniques such as solid phase microextraction, monotrap, and a dynamic headspace sampling system have been used for emitted volatiles [64]. Earlier studies of terpenoid metabolite profiling from different plant species are as follows: twenty-three terpenes were recently analyzed using different accelerated solvent extraction (ASE) methods [65]. In a comprehensive analysis of terpenes and terpenoids in medicinal cannabis biomass, a total of 49 distinct individual compounds were detected [52]. Using 12 cannabis samples, 30 terpene compounds were detected in another study [66]. Nguyen et al. used a robust testing method to quantify 30 selected terpenoids in dry plant materials, with 30 monoterpenoids and sesquiterpenoids quantified [53]. Using a solid-phase microextraction method, 28 terpenes were identified and quantified in Muscat grape cultivars [67]. In another study of *Citrus medica* (finger citron), a total of 62 volatiles were detected, among which monoterpenoid limonene and *γ*-terpinene were most abundant [68]. The different developmental stages of eight Artemisia plants were analyzed by GC-MS profiles, which consisted of 40–90% of monoterpene and sesquiterpene derivatives [54]. Another study comparing the terpene profiles of four important fruits (gooseberry, crab apple, cherry silver berry, and scarlet haw-thorn) identified 79 terpenoid compounds [69]. Three different extraction methods were used, and a total of 81 volatile compounds were identified in *Exocarpium citri* Grandis, around 58% of which were terpenes [70]. Similarly, different methods were tested on *Ocimum basilicum* leaves and 18 terpenoid compounds were identified [71]. Ma et al. performed metabolite profiling on three different ginger (*Zingiber officinale*) lines containing 102 compounds, among which 29 monoterpenoids and 47 sesquiterpenoids were identified [72]. Table 2 shows the metabolite profiling and identified terpenoid compounds in the different plant species.

## 4. Metabolic Engineering of Terpenoids in Plants 

Changing the availability of precursors is one method used to control terpenoid levels in plants. It is possible that altering the terpenoid precursor pool alone is not enough to elevate levels of target terpenoids, and that simultaneously engineering the downstream genes would also be needed. In metabolic engineering, the availability of precursors is a key concern. The level of an isoprenoid precursor that is limiting for the synthesis of terpenoids is likely to vary depending on the plant tissue, species, and physiological condition [16]. A previous study of the metabolic engineering of monoterpenoids focused on producing heterologous monoterpenes in spearmint, and the knocking down of limonene synthase resulted in a huge reduction of limonene and carvone synthesis, while RNAi plants showed an increased sesquiterpenoid level [73]. The overexpression of geranyl diphosphate synthase small subunit 1(GPS SSU1) in *Listea cubeba* (Lour) plants showed a significant increase in monoterpene levels [74]. Two potential genes in the MEP pathway, namely, DXS and DXR, were cloned and developed in transgenic tobacco plants, which resulted in an increased content of monoterpenoid and sesquiterpenoid linalool and caryophyllene, respectively [75]. The co-expression of *Solanum lycoperscum* DXP, *Arabidopsis thaliana* GPS1, and Mentha X piperita GPS SSU through transient expression in *Nicotiana benthamiana* plants enhanced the production of monoterpenes such as limonene, linalool, alpha/beta-pinene, and myrcene [76]. The monoterpene key enzyme terpene synthase (TPS) subfamily was expressed in *Osmanthus fragrans* and the transient expression of leaves exhibited a high level of linalool and trans-β-ocimene, hence TPS played an important role in monoterpene production [77]. The development of transgenic *Mentha spicata* by *Agrobacterium tumefaciens* mediated transformation with isopentyldiphospahte (IPP) isomerase, and limonene synthase gene resulted in high levels of terpenoid production [78]. The overexpression of HMG CoA reductase in *Lavandula angustifolia* resulted in high levels of linalool production [79]. In another study, snapdragon (*Antirrhinum majus*) GPPS-SSU was overexpressed in tomato fruits, resulting in the production of monoterpenes including geraniol, geranial, neral, citronella, and citronellal [80]. Lucker et al. found that the production level corresponding to three different monoterpene synthases was high in transgenic tobacco plant flowers exhibiting three separate monoterpene synthases, but that the expression of endogenous linalool production was not affected [81]. In *Arabidopsis*, to produce monoterpenes, the strawberry gene nerolidol synthase 1 was used, and this resulted in the formation of linalool; this gene also encodes the dual function for the production of monoterpenes and sesquiterpenoids [14]. In another study, *Perilla frutescens* limonene synthase was introduced to tobacco plants, and limonene formation was detected in the plastids and cytosol of transgenic plants [82]. In petunia, the overexpression of *S*-linalool synthase resulted in the monoterpene production of newly formed linalool [10]. The overexpression of the *S*-linalool synthase gene in tomato (*Lycopersicon esculentum*) resulted in high levels of monoterpenes [11]. The results of many of the studies reported to date suggest that, in general, the direct precursor for monoterpene biosynthesis (i.e., GPP) is largely available to introduced monoterpene synthases.

The metabolic engineering and overexpression of sesquiterpenoids is limited to certain sources; β-caryophyllene synthase from *Artemisia annua* was introduced to a viral vector and transferred into *Agrobacterium*, which was then agroinfiltrated into *N. benthamiana* leaves and produced 26.5 mg of β-caryophyllene [83]. In an in vitro regeneration study, sesquiterpene cyclase was transformed in the medicinal plant *A. annua*, and resulted in a 30% atremisinin content [84]. The co-expression of the parthenolide pathway candidate genes was reconstituted by transient co-expression in *N. benthamiana*, and up to 1.4 µg of the final product was produced [85]. The key enzyme amorpha-4,11-diene synthase was transformed into *Agrobacterium* and agroinfiltrated into *N. benthamiana*, which resulted in various sesquiterpene products [86]. Co-expression with different plant-specific genes, such as feverfew germacrene A synthase (GAS), chicory germacrene A oxidase (GAO), and chicory costunolide synthase (COS), was reconstituted and agroinfiltrated into *N. benthamiana*, and results showed 60 ng g^−1^ of costunolide production [87]. Furthermore, different plant species have been used successfully to produce valuable sesquiterpenoid compounds using plant suspension cell culture technology [88,89]. Hitherto, five mevalonate and artemisinin pathway genes were expressed in tobacco plant cell cultures using a single vector, yielding 0.48–6.8 μg/g of artemisinin [90]. Similarly, constructing four genes (amorpha-4,11-diene synthase, amorphadiene monooxygenase, aldehyde ∆ (13) reductase, and aldehyde dehydrogenase) in *N. tabacum* leaf cells resulted in 0.01 mg/g of artemisinic alcohol [91]. Likewise, five artemisinin biosynthesis genes were transferred into *Physcomitrella patens*, which produced 0.21 mg/g of artemisinin after three days of cultures [92]. Table 3 summarizes the overall documented list of metabolic engineering of mono-terpenoids and sesquiterpenoids targeting genes and upregulated metabolites.

## 5. Synthetic Biology of Terpenoids

Synthetic biology is defined as the ‘design and building of novel biological components such as enzymes, genetic circuits, or restructuring of existing biological system’. Various monoterpenoids and sesquiterpenoids were synthesized over the last decade using engineered bacteria and yeast [93,94]. The most well-known example of synthetic biology-based high value chemical production is artemisinic acid, the precursor of the antimalarial drug arteminisin, which was synthesized in engineered *Escherichia coli* and baker’s yeast, *Saccharomyces cerevisiae*, following ten years of optimization [95,96]. Furthermore, a specialized limonene and perillyl alcohol manufacturing system was established in *E. coli* by co-expression of heterologous, codon-optimized, *Staphylococus aureus*, and *S. cerevisiae* MVA pathway genes into *E. coli* with *Abies grandis* GPP synthase and *Mentha spicata* limonene synthase genes. Optimum gene regulation and growing circumstance resulted in a 400 mg/L limonene titre [97]. However, alternative strategies in *E. coli* focused on the MEP pathway genes DXS, and because IDI was overexpressed, the resulting strains provided a poor titre of 35.8 mg/L limonene [98]. Similarly, using *Yarrowia lipolytica* yeast, sesquiterpenes (+)-nootkatone was synthesized by heterologous co-expressing genes such as valencene synthase, nootkatone synthase, and NADPH-cytochrome P450 reductase, resulting in 978.2 μg/L (+)-nootkatone [99]. As an application, most synthetic biology research on monoterpenoids and sesquiterpenes has focused on the high level of production in few compounds. However, a number of other monoterpenoids and sesquiterpenes have been produced in *E. coli* or yeast, and other microbial systems by assembling and optimizing biosynthetic pathways that contain a heterologous MVA or MEP pathway, as well as GPP and FPP. 

Moreover, the field of synthetic biology in plants is still in its infancy. Synthetic biology research on plants needs reliable and effective techniques for compiling and transforming multi-component DNA constructions, such as a promoter and terminator. Reporter gene fusion is also a regular task in this field. Hence, optimizing this procedure is likely to result in significant productivity improvements. Traditionally, the required DNA construct has been designed using the restriction of endonuclease-mediated cleavage together with T4 DNA ligase-mediated joining. However, this approach takes a long time, and reliability along the sequence is not good for a large number of structures in a very efficient assembly [100,101,102]. Synthetic biology, which was inspired by engineering and mechanical assembly lines, uses standardized components to construct genetic creations. By using a Type IIS cloning system such as Golden Gate, MoClo, GoldenBraid, Loop Assembly, Fragment exchange, and others strategies for assembling synthetic constructs, high-throughput combinatorial libraries of synthetic constructs were easily constructed [103,104]. Internal occurrences of the recognition sequence are a constraint of Golden Gate Cloning. Type IIS restricted enzymes are employed in Golden Gate Cloning. Such enzymes only cut at a single site beyond their recognition-binding site sequence. BsaI detects the sequence 5′-GGTCTC-3′. Each fragment that will be integrated into the target vector is bounded by BsaI sites in the usual cloning process. [105]. Many of the reports included in the parts kit are also applicable to non-plant species. Such parts apply directly to multiple systems. Combinatorial pathway reconstruction includes all of the vector backbones and sequences needed for domesticating additional sequences and assembling them into single and multigene binary constructs [106]. These types of constructs are widely utilized in synthetic biology, and are a vital part of combinatorial bioengineering in plants. Additionally, with the emergence of CRISPR-Cas9 technology, large scale genome editing has become more feasible and affordable. All of these technological developments have largely eliminated the bottleneck associated with multi-gene transfer in plants. The development of new transformation technologies to generate stable transformed vector and cell lines with multiple constructs should be a high priority for commercial success in sustainable plant terpenoid metabolite production using synthetic biology approaches. Together, these tools will improve the bio-industrial production of monoterpenoids and sesquiterpenoids. 

## 6. Terpenoids Pharmacological Activity

Terpenoids have been extensively utilized as raw materials in medicines because they have anti-inflammatory, antitumor, antiviral, antibacterial, and antimalarial properties, and they have the ability to increase transdermal absorption, prevent and cure cardiovascular disease, and exhibit hypoglycemic properties. 

### 6.1. Monoterpenoids

Monoterpenes are C10 terpenoids produced from plastids that have high volatility. As a result, they are often found in plant essential oils. Below, we describe the three major classes of monoterpenoids (linalool, limonene, and alpha-pinene) with well-documented pharmacological properties.

#### 6.1.1. Linalool

Linalool (C_10_H_18_O), also known as 3,7-dimethyl-1,6-octadien-3-ol, is an acyclic monoterpene tertiary alcohol found in the essential oils of various plant species [107]. Linalool is the primary element of various essential oils, which have been shown to have a range of biological activities, including antibacterial as well as anti-plasmodial properties [108]. Linalool has been shown to have anti-hyperalgesia, anti-inflammatory, and anti-inociceptive properties in a variety of animal models [109]. The anti-oxidant properties of *Cinnamomum osmophloeum* (Lauraceae) oil scavenged the DPPH radical (IC50 value: 29.7 g/mL), and this action was linked to the main component linalool, which made up 73% of the whole [110]. In the traditional medicinal system, different species of linalool and linalyl acetate are utilized to alleviate symptoms and treat many chronic and acute ailments [111]. Linalool-producing species have been shown to have anti-inflammatory properties and a peripheral analgesic impact [112,113]. 

#### 6.1.2. Limonene

Limonene (C_10_H_16_)—(R)-4-Isopropenyl-1-methylethenyl-cyclohexene is a monocyclic monoterpene found in a variety of plants and a common essential oil component of aromatic plants [114]. Limonene has significant uses in cosmetics, soft drinks, and a variety of flavoring products. It has received increased interest due to its anticancer, antimicrobial, toxicity, and antiparasitic activities, among others [114]. Dabbah et al. reported the antimicrobial activity of pure limonene and the oil to be extremely effective when comparing the inhibitory impact of the essential oils from the fruits of lemon, orange, mandarin, grapefruit, and d-limonene [115]. According to Keinan et al., the anti-oxidant activity of limonene may readily saturate the pulmonary membrane, and therefore protect lung cells against both endogenous and exogenous ozone [116]. 

#### 6.1.3. Alpha-Pinene

Pinene (C_10_H_16_)—(1S,5S)-2,6,6-Trimethylbicyclo (3,1,1) hept-2-ene—is a bicyclic monoterpene found in essential oils of pine (coniferous trees) [117]. Pinene has a wide range of biological activities, which means it has a wide range of applications, including in fungicidal agents, flavorings, perfumes, and antiviral agents [118]. Due to its toxic effects on membranes, pinene is also employed as an antibacterial agent [119]. Furthermore, pinene has been shown to suppress breast cancer and leukemia [120]. Moreover, pinene has potential as a natural medicine; for example, it is particularly flexible in the synthesis of polymers [121].

#### 6.1.4. Others 

Several *p*-menthane monoterpenoids of pharmacological relevance are found in the genus Mentha (Lamiaceae). (−)-Menthol, a key component of the essential oil of peppermint (Mentha × piperita) since the 1950s, has been recognized to serve as a full agonist of the CMR1 (Cold and Menthol Receptor 1) [122]. Cannabinoids were first used to describe a group of prenylated phenolic substances found in *Cannabis* spp. (Cannabaceae) but now comprise any ligand capable of interacting with human cannabinoid receptors; specifically, this includes endogenously-generated cannabinoids with no molecular resemblance to their plant-derived, terpene phenolic counterparts [123]. Cannabinoids, like p-menthane monoterpenoids in the Lamiaceae, accumulate in glandular trichomes, while cannabinoid-rich trichomes in the genus *Cannabis* are mainly found in the calyces and bracts of female flowers [124].

### 6.2. Sesquiterpenoids

Sesquiterpenoids have been reported to exhibit many pharmacological effects, including anti-inflammatory, antiviral, antibacterial, antifungal, antifeedant, antinociceptive, antileshmanial, and cytotoxic effects; they also exhibit lymphocyte proliferation and hydroxy radical scavenging. Below, we present three therapeutically important plant sesquiterpenoids. 

#### 6.2.1. Costunolide

Costunolide (CT) is a well-known sesquiterpene lactone of the germacranolides class. It is a white crystalline powder with the chemical formula C_15_H_20_O_2_. This chemical was first isolated from the root of costus (*Saussurea lappa* Clarke), and later from lettuce (*Aucklandia lappa*) and many other plant species [125]. Several studies have shown that CT has effects on anitbladder cancer [126], ovarian cancer [127], leukemia [128], and prostate cancer [129]. It was also reported that costunolide inhibits angiogenic responses by blocking the angiogenic factor signaling pathway and microtuble-interacting activity of costunolide [130,131].

#### 6.2.2. Parthenolide

Parthenolide (C_15_H_20_O_3_) is considered to be one of the main active components in feverfew, accounting for many of the plant’s biological characteristics [132]. Recently, feverfew has been widely used for the prophylaxis of migraine headaches, relief of the pain and inflammation associated with arthritis, and the treatment of psoriasis [133]. As well as occurring in feverfew, parthenolide is the major anti-inflammatory component of tansy (*Tanacetum vulgare*). In animal studies, parthenolide significantly alleviated carrageenan-induced paw oedema when administered orally, with a stronger effect following intraperitoneal administration [134].

#### 6.2.3. Trans-Caryophyllene

Trans-caryophyllene has been reported to possess many pharmacological effects. For example, it displays antimicrobial [135] and analgesic activity [136], and has a well-documented anti-inflammatory activity [137,138]. Additionally, trans-caryophyllene is an effective treatment for intestinal smooth muscle, blocking the electromechanical and ablepharmacochemical excitation–contraction coupling [139]. Those activities mean it is considered as a potential anti-spasmodic agent in tracheal smooth muscle.

#### 6.2.4. Others

On the basis of dozens of carbon skeletons, sesquiterpene synthases act on FDP to produce hundreds of sesquiterpene hydrocarbons and alcohols. Clove, ginger (gingerol), rosemary (-caryophyllene), cannabis, sandalwood (-santalene), rain (geosmin, a bacterial sesquiterpene), and sandalwood (-santalene) are just a few examples that are responsible for flavors and fragrances. Under normal conditions, they are the heaviest of the volatile terpenes (heat is usually required to generate gases from diterpene hydrocarbons). Geraniaceae, Lamiaceae, Myrtaceae, Rutaceae, Gingeraceae, and Cannabaceae are among the plant families that generate sesquiterpene volatiles. It is widely acknowledged that such essential oils are used in traditional herbal treatments, including Ayurvedic and aromatherapy medicine [41]. Wormwood (*Artemisia annua* L., also known as qinghaosu, in family Asteraceae) is a Chinese plant that produces the sesquiterpene endoperoxide artemisinin, which has been proven to be efficient in the treatment of malaria [140]. It is more effective than conventional antimalarials, such as quinine, against a broader range of apicomplexan parasite life cycle stages [141].

## 7. Conclusions and Future Perspectives 

This review mainly focused on the metabolic engineering and synthetic biology caused by the overexpression of terpenoid compounds in plants due to their diverse and biologically significant uses for humans. Over the last few years, research has taken advantage of advances in genomics, transcriptomics, and metabolomics, which has resulted in a greater understanding of the pathways and regulatory mechanisms involved in the biosynthesis of specialized terpenoids. Furthermore, the identification of regulatory factors and gene clusters involved in the biosynthesis of terpenoids in various plant species has provided a means to improve the biosynthesis of specialized terpenoids. Despite their several chemical constituents, monoterpenoids and sesquiterpenoids were investigated in detail. Particular compounds were overexpressed using single-construct vectors. Future studies should focus on the combination of multiple biosynthesis pathway genes constructed in single cloning vectors and agro-infiltrated in models, as well as original plants which are essential for the mass production of terpenoids in plants over a short period of time. 

## Figures and Tables

**Table 1:** List of GC-MS column and oven program for terpenoid analysis.

Instrument	Column Name	Dimension (Length, Inner Diameter & Thickness)	Oven Program	References
GC-MS	DB-5MS-DG, DB-17, VF-35	30 m × 0.25 µm ID × 0.25 µm, 30 m × 0.25 µm ID × 1.0 µm	60 °C (2 min), Ramp: 5 °C/min to 200 °C	[52]
GC-MS	TG-624 SilMS	30 m × 0.25 mm ID × 1.4 µm	60 °C (30 s), Ramp 1: 15 °C/min to 130 (3 min); Ramp 2 5 °C/min to 140 °C (1 min); Ramp 3: 22° C/min 280 °C (3 min)	[53]
GC-MS	HP-5	30 m × 0.25 µm ID × 0.25 µm	50 °C (2 min), Ramp 1: 5 °C min to 180 °C, Ramp 2: 20 °C/min to 270 °C	[54]
GC-MS	Rxi-624 Sil MS	30 m × 0.25 mm ID × 1.4 µm	80 °C (1 min), Ramp 1: 12 °C/min to 150 (1 min); Ramp 2 9 °C/min to 250 (1 min)	[55]
GC-FID	ZB-5 PLUS^TM^	20 m × 0.18 mm ID × 0.36 µm	Ramp 1: 35 °C to 105 °C 10 °C/min to 205 °C Ramp 2: 15 °C/min to 360 °C Ramp 3: 35 °C/min for 1.9 min	[56]
GC-MSD	DB-HeavyWax	30 m × 250 µm ID × 1.4 µm	50 °C (0.75 min), Ramp 1: 80 °C (0 min); Ramp 2: 240 °C (5 min)	[57]
GC-FID	VF-624 ms	60 m × 0.32 mm ID × 1.8 µm	90° C (1 min), Ramp 1: 15 °C min to 181° C (3 min)	[58]
GC-MS	Elite-5	30 m × 0.25 µm ID × 0.25 µm	100 °C (5 min), Ramp 1: 20 C/min (200° C), Ramp 2: 10 °C/min (270 °C)	[59]

**Table 2:** Identification of terpenoid metabolite compounds and analytical methods used.

Source	Instrument *	Method	Identified Terpenes	References
Cannabis	GC-MS	ASE	23	[65]
Cannabis	GC-MS	Headspace, SPME and Liquid injection	49	[52]
Cannabis varieties	GC-MS	HS-SPME	30	[66]
Cannabis	GC-MS	Headsapce	30	[53]
Muscat grape	GC-MS	HS-SPME	28	[67]
Finger Cirton	GC-MS	SPME	62	[68]
Fourteen Compositae plants	GC-MS	n-hexane	213	[54]
Goose berry, crabapple, cherry silver berry, scarlet hawthornq	GC × GC-TOF-MS	SPME	79	[69]
*Exocarpium citri* Grandis	GC-MS	SP, HS-SPME & solvent extraction	81	[70]
Basil & Tobacco	GC-MS	SP, Ultrasound-Assisted	18	[71]
Zinger	GC-MS/LC-ESI-MS	MeOH	102	[72]

* GC-MS—Gas chromatography-mass spectrometry; GC-TOF-MS—Gas chromatography time of flight-mass spectrometry; LC-ESI-MS—Liquid chromatography electrospray ionization mass spectrometry; ASE—Accelerated solvent extraction; HS-SPME—Headspace solid-phase microextraction; SPME—Solid-phase microextraction; SP—Solid phase.

**Table 3:** Reports on the metabolic engineering of monoterpenoids, sesquiterpenoid targeted genes, their derivatives, as well as their precursors and upregulated metabolites are listed.

Source	Species	Targeted Genes	Up/Downregulated	References
	**Monoterpenoids (C15)**			
Mentha	*Mentha spicata*	Limonene synthase	Incresead in sesquiterpenoid	[73]
Lour	*Litsea cubeba*	Geranyl diphosphate synthase small subunit 1	Increase in monoterpene content	[74]
Lilium	*Lilium* “Siberia”	1-deoxy-d-xylulose-5-phosphate synthase, 1-deoxy-d-xylulose-5-phosphate reductoisomerase	Linalool (mono), Caryophyllene (sesqui)	[75]
Mentha X piperita	*Nicotiania benthamiana* & *Nicotiania tabacum*	Geranyl diphosphate synthase small subunit	(−) Limonene, (−)-Linalool, (−)-β-pinene, (−)-α-pinene, Myrcene	[76]
Sweet osmanthus	*Osmanthus fragrans*	Terpene synthase	β-linalool, trans-β-ocimene, α-farnesene	[77]
Mentha	*Mentha spicata*	IPP isomerase & limonen synthase	1,8-cineole, linalool, camphor, terpinene, lomonene, borneol, safranal, geraniol, thymol, 1-α-terpineol, methyl eugenol, menthone, menthol-isomer, thymol, piperitone	[78]
English lavender	*Lavandula angustifolia*	Linalool synthase, HMG-CoA reductase	Linalool	[79]
Snapdragon	*Antirrhinum majus*	Geranyl diphosphate synthase small sub unit	Increase in monoterpene and sesquiterpene content	[80]
Tobacco	*Nicotiania tabacum*	ϒ-Terpinene synthase	ϒ-Terpinene, limonene, β-pinene and side products	[81]
Arabidopsis	*Arabidopsis thalina*	Linalool/nerolidol synthase	Linalool, hydroxylated and glycosylated linalool	[14]
Tobacco	*Nicotiania tabacum*	Limonene synthase	Limonene	[82]
Petunia	*Petunia hybrida*	Linalool synthase	Linalool glycoside	[10]
Tomato	*Lycopersicon esculentum*	Linalool synthase	Fruit-Linalool & hydroxylated linalool	[11]
	**Sesquiterpenoids (C15)**			
Sweet wormwood	*Nicotiania benthamiana*	β-caryophyllene synthase	β-caryophyllene	[83]
Sweet wormwood	*Artemisia annua*	Sesquiterpene cyclase	Atremisinin	[84]
Feverfew	*Tanacetum parthenium*	Parthenolide synthase	Parthenolide	[85]
Sweet wormwood	*Nicotiania benthamiana*	Sesquiterpene synthase	Amorpha-4,11-diene & epi-cedrol	[86]
Lettuce	*Lactuca sativa*	Costunolide synthase	Costunolide	[87]
